# Early Onset Preeclampsia Diagnosis Prior to the 20th Week of Gestation in a Twin Pregnancy Managed via Selective Reduction of an Intrauterine Growth Restriction Fetus: A Case Report and Literature Review

**DOI:** 10.3390/diagnostics10080531

**Published:** 2020-07-29

**Authors:** Anastasios Konstantopoulos, Konstantinos Sfakianoudis, Mara Simopoulou, Adamantia Kontogeorgi, Anna Rapani, Sokratis Grigoriadis, Agni Pantou, Nikolaos Bathrellos, Alexandros Grammatis, Konstantinos Pantos

**Affiliations:** 1 Centre for Human Reproduction, Genesis Athens Clinic, 14–16, Papanikoli, 15232 Athens, Greece; drtkon@hotmail.com (A.K.); sfakianosc@yahoo.gr (K.S.); agni.pantos@gmail.com (A.P.); nikosbathrellos@yahoo.gr (N.B.); alexgrammatis@gmail.com (A.G.); info@pantos.gr (K.P.); 2 Laboratory of Experimental Physiology, Department of Physiology, Medical School, National and Kapodistrian University of Athens, Mikras Asias 75, 11527 Athens, Greece; ad.kontogewrgi@gmail.com (A.K.); rapanianna@gmail.com (A.R.); sokratis-grigoriadis@hotmail.com (S.G.)

**Keywords:** early onset preeclampsia, twin pregnancy, selective embryo reduction, live birth

## Abstract

A single, healthy, 44-year-old perimenopausal woman pursuing a pregnancy, employed donor embryos, resulting to a dichorionic diamniotic twin pregnancy. In the 18th week of gestation severe symptoms indicated early onset preeclampsia reporting severe hypertension (BP 180/90 mmHg), intense headaches and nausea as well as elevated 24-h urine protein levels (1.5 g/day). Concurrently diagnosis of an IUGR fetus was concluded. Standard pharmaceutical administration for treating preeclampsia was ordered. Persistence of symptoms indicated recommendation for pregnancy termination, however the patient opted against this. Selective embryo reduction was performed as the last resort prior to pregnancy termination. Following selective reduction the headaches and nausea were successfully subdued and the patient’s blood pressure was adjusted (mean BP 130/80 mmHg). This enabled further progression of pregnancy for an impressive 11 week-period, and a live birth on the 30th week. To conclude, only a few rare cases have been reported with diagnosis of early onset preeclampsia prior to the 20th week mark and none report live births. Albeit termination of pregnancy was recommended, the management of selective reduction of the IUGR fetus enabled successful treatment of preeclampsia coupled by a live birth of a healthy infant without any perinatal or postnatal complications reported.

## 1. Introduction

Preeclampsia is a term employed to describe the appearance of proteinuria and hypertension during gestation and it affects 5% of pregnancies [1]. The criteria for diagnosing preeclampsia have not been subject to modification over the past few years [2]. In order to classify a patient as preeclamptic, certain criteria should be fulfilled including a protein concentration ≥30 mg in two or more urine samples 5 h or up to 7 days apart, diastolic blood pressure ≥90 mmHg in ≥2 measurements, systolic blood pressure >140 mmHg in ≥2 measurements, and proteinuria ≥30 mg/day in a 24-h urine sample. In clinical practice, several risk factors associated with preeclampsia have been identified, namely obesity, diabetes, maternal age >35, chronic hypertension, kidney disease, previous preeclampsia, twin pregnancy, molar pregnancy, fetal congenital abnormality and even high altitude since it attributes to lower uterine artery blood flow, increased levels of placental hypoxia and shorter uterine artery diameter [3,4,5]. Complications constitute a major issue encountered in cases of preeclampsia with an 8% complication rate described in the western world, rendering preeclampsia a major factor of maternal and fetal mortality worldwide [6,7]. Interestingly, 16–18% of maternal perinatal deaths and up to 40% of fetal and neonatal deaths are attributed to cases of preeclampsia [8].

Commonly, two types of preeclampsia complications are encountered, including obstetric and non-obstetric events. Obstetric complications include—albeit not limited—to intrauterine growth restriction (IUGR), preterm delivery, HELLP syndrome with increased risk of eclampsia and liver rupture, and fetal death (IUFD) [8]. Non-obstetric complications involve pulmonary edema, liver failure (American College of Obstetricians and Gynecologists and Task Force on Hypertension in Pregnancy, 2013), disseminated intravascular coagulation (DIC), acute renal failure, heart failure, strokes or posterior reversible encephalopathy syndrome that could be classified as cerebrovascular incidents and partum cardiomyopathy. Despite the fact that these complications may not affect the viability or growth of a fetus, it should be highlighted that they hold the potential to trigger maternal organ dysfunction [9]. The mortality and morbidity percentages of preeclampsia are similar to the ones of eclampsia and the occurrence of the disease is 1.5–10 out of 100,000 deliveries [10]. It becomes evident that preeclampsia may induce severe consequences during pregnancy, nonetheless diagnosing preeclampsia in early stages of pregnancy may improve the perinatal outcome for both the mother and the fetus.

Early onset preeclampsia is defined as diagnosis of preeclampsia prior to the 34th week mark [11]. Early onset preeclampsia affects approximately 4 per 1000 pregnancies in nulliparous women and it is associated with adverse perinatal outcomes and high perinatal mortality [12]. Moreover, it has been voiced that early onset preeclampsia is also associated with increased risk of stillbirth, reporting 11.6 stillbirths per 1000 pregnancies in the 26th week of gestation. However, the risk for stillbirth is significantly reduced as pregnancy advances [13]. Impressively, studies have demonstrated that incidents of preeclampsia could also appear as early as prior to the 20 weeks milestone during gestation [14]. Nonetheless, only a few live births have been reported for cases where preeclampsia has been diagnosed prior to the 20th week of gestation. Moreover, preeclampsia may be present despite the absence of any symptoms indicating proteinuria or hypertension [15]. In the occurrence of antiphospholipid syndrome or partial molar pregnancy with triploidy, along with the detection of early onset preeclampsia the term atypical preeclampsia is employed [16,17,18,19]. The cases where early onset preeclampsia prior to the 20th week mark is diagnosed in the absence of the aforementioned disorders are extremely rare in literature, with only six cases published hitherto [14,20,21,22,23,24]. It should be noted that none of the published studies has reported a live birth following management of early onset preeclampsia, indicating the knowledge gap in efficiently addressing and treating preeclampsia symptoms while ascertaining a positive pregnancy outcome. This very fact renders this study timely and essential.

This study aims to provide data regarding a rare case report of a patient with symptoms of early onset preeclampsia during a dichorionic diamniotic twin pregnancy in the 18th week of gestation. This case was managed via selective embryo reduction on the IUGR fetus, resulting to an impressive improvement of the clinical symptoms related to preeclampsia. The authors herein further provide a review synthesis of published cases similarly reporting on the extremely rare phenomenon of early onset preeclampsia prior to 20th week of gestation. This documentation further contributes to the literature and standing management options by providing data on successful preeclampsia management via selective embryo reduction.

## 2. Case Report Description

A single, healthy 44-year-old, Caucasian, perimenopausal woman, gravida zero, parity zero (G0P0), pursuing a pregnancy was subjected to assisted reproduction treatment employing donor embryos obtained from a fresh assisted reproduction treatment (ART) cycle. An embryo donation cycle pertains to employment of oocytes yielded following an oocyte retrieval procedure from an oocyte donor and sperm originating from a sperm donor employed within the context of an ART cycle.

Patient’s body mass index (BMI) was 25.4 kg/m^2^ and her medical history was free of smoking, infections, sexually transmitted diseases, autoimmune disorders, diabetes mellitus and chronic hypertension. Her familiar medical history was also free of pregnancy hypertensive disorders, including preeclampsia. The patient was presenting with menstrual cycle irregularities, elevated follicle stimulating hormone (FSH) and luteinizing hormone (LH) levels (34.31 mIU/mL and 20.72 mIU/mL, respectively) and significantly reduced estradiol levels (12.0 pg/mL). Considering that the patient was of advanced maternal age and of diminished ovarian reserve, she opted for embryo donation as the last resort to pursuing a pregnancy.

Prior to ART treatment the woman was subjected to extensive clinical and biochemical investigation in the context of screening for any possible underlying pathologies as certain conditions are typically evaluated prior to proceeding to treatment. Following examination, no signs of hypertension, thyroid disease, diabetes mellitus, antiphospholipid syndrome, thrombophilia or any other thrombophilic disorder were noted. Regarding the embryo donation cycle’s characteristics, ten oocytes were obtained from a 26 years old, healthy oocyte donor, following controlled ovarian stimulation, employing the standard gonadotropin-releasing hormone (GnRH) long agonist protocol [25]. Oocytes were fertilized via intracytoplasmic sperm injection (ICSI), employing a sperm donor sample originated from a healthy male donor. In total, 8 blastocyst stage embryos were obtained. Embryo transfer was performed employing two top quality blastocysts (5AA) according to Gardner’s grading system [26]. At this point it is of paramount importance to note that robust data, provided from large randomized controlled trials as well as from systematic reviews and meta-analysis, indicate that the selective single embryo transfer (eSET) is an effective method to reduce the risk of multiple births, compared to double embryo transfer, without compromising the pregnancy outcomes [27,28,29,30,31,32]. Considering the aforementioned, eSET is strongly suggested for all patients [27,28,29,30,31,32]. Our patient was advised to proceed with eSET. Nonetheless, following consultation, it was the patient’s desire to procced with a double embryo transfer aiming to increase the possibility for achieving a pregnancy albeit acknowledging the possibility of a twin pregnancy and the possible risks entailed for a twin pregnancy. Please kindly note that respective legislation in Greece permits a double embryo transfer even in patients below the 35-year-old mark extending to oocyte and embryo donation cycles. For these reasons, and in spite of the consultation the patient received, a double embryo transfer was performed.

Four weeks following recipient’s last menstruation, a TVUS was performed revealing the existence of two sonolucent sacs surrounded by an echogenic ring of chorionic villi, indicating the successful implantation of the two blastocysts that were transferred. Two weeks later, at the end of the 6th week, clinical pregnancy was confirmed via TVUS indicating two independent gestational sacs with fetal heartbeat. Following the confirmation of dichorionic diamniotic twin pregnancy, the patient returned to their place of permanent residence being a small provincial town. The patient opted to continue pregnancy monitoring at the local health care center coupled by the supervision provided by a Fetal Medicine specialist in the region. Following the confirmation of clinical pregnancy and until the 12th week mark, the pregnancy progressed normally without any complications.

On the 12th week of gestation, the patient underwent first trimester nuchal translucency transabdominal scan revealing a dichorionic diamniotic twin pregnancy with measurements consistent with the gestational age estimated at the time. Both embryos presented with normal Crown Rump Length (CRL) as well as normal nuchal translucency (NT) measurements [33,34]. For both embryos a visible nasal bone was observed. There was no evidence or signs of any anatomical defects for either of the fetuses. Along with NT, a Doppler ultrasound of the uterine artery was also performed indicating Mean Uterine Artery PI (UTPI) equal to 2.2 (1.539 MoM). Employing the Astraia Obstetrics software for First Trimester Risk calculation, the specialist who performed the NT measurements and transabdominal ultrasound assessed the preeclampsia risk while considering maternal characteristics and medical history following examination of blood pressure and blood flow in the uterus. Considering these assessments, the patient was classified to be at increased risk of developing preeclampsia prior to 37 weeks of gestation. To prevent the onset of preeclampsia, a low dose of aspirin (150 mg per day) was prescribed from that point onwards.

At the same time, a double marker test was also performed via the evaluation of serum PAPP-A levels as well as of serum free beta-hCG levels. Considering the oocyte donor’s age, data indicated that the fetus was at a low risk of fetal aneuploidy. Therefore, prenatal diagnosis testing employing amniocentesis was not recommended [35]. Nonetheless, the patient proceeded with non-invasive prenatal diagnosis testing, which did not detect increased risk for aneuploidy for either of the embryos. Moreover, non-invasive prenatal diagnosis indicated the sex of the fetuses one identified as being female and the other as male.

During the 14th week of gestation, the patient referred to the local health care center, complaining of mild dizziness and headache. Based on the physical examination that followed, the patient’s blood pressure (BP) was 130/80 mmHg. Trans abdominal ultrasonography was performed indicating that both fetuses were consistent to the gestational age of 14 weeks and 1 day for embryo 1 and 14 weeks and 3 days for embryo 2, respectively. The attending physician advised the patient to report to an obstetric hospital in order to focus on effectively addressing the hypertension. Nonetheless, despite clear guidance and consultation, the patient never attended the obstetric hospital. Thenceforth, the patient failed to present at her next scheduled appointment both at the local health care center, as well as at the fetal medicine specialist and follow-up for the subsequent 4 weeks was lost.

During the 18th week of gestation (18 weeks and 4 days) the patient referred to the local health care center again, with severe hypertension (BP 180/90 mmHg) coupled by intense headaches and nausea. The patient was immediately admitted to our clinic and an evaluation of differential diagnosis was initiated. The patient had no history of hypertension, renal disease, diabetes mellitus, or illicit drug use, and no familiar history of preeclampsia. Based on the laboratory exams, the complete blood count, clotting, and liver function (SGOT 25 U/L and SGPT 55 U/L) were normal. Renal function was mildly reduced with creatinine levels at 0.85 mg/dL and an estimated glomerular filtration rate (eGFR) of 77 mL/min. The patient’s 24-h urine protein was 1.5 g/day, serum albumin was 3 g/dL, globular 1.1 g/dL and total proteins 4.94 g/dL. Increased blood pressure levels, combined with the elevated 24-h urine protein, along with the clinical manifestations, established the diagnosis of preeclampsia. Based on NICE guidelines for preeclampsia management, methyldopa (500 mg × 3 per day) was administrated as the pharmaceutical therapeutic regime of choice.

During the same gestational week, a transabdominal pregnancy scan of the fetuses was also performed. Ultrasound revealed a 31% weight discrepancy between the embryos. The smaller fetus identified as male and presented herein as Embryo 2, presented with oligohydramnio along with an estimated weight of 240 g (<3rd centile) and negative end diastolic flow in umbilical artery. Selective IUGR was diagnosed and patient’s hospitalization was recommended highlighting the urgency for regulating her blood pressure levels. Taking into account both assessments, it was evident that the pregnancy was classified as both of an increased maternal as well as fetal risk, and considering the possible severe obstetrics and non-obstetrics complications, termination of pregnancy was recommended.

Despite the selective IUGR diagnosis and the recommendation for pregnancy termination, the patient denied recommendation and persisted on maintaining the pregnancy. Close monitoring of the clinical and biochemical status of the patient was undertaken. In case the symptoms did not subside, a termination of the pregnancy would be imperative and inevitable. The patient remained hospitalized under methyldopa treatment. Over the course of the next five days, there were no signs of improvement in the patient’s persistent symptomatology in regard to proteinuria and the elevated blood pressure levels. Blood pressure was not regulated within the normal ranges and the symptoms of nausea and headaches were not mitigated. A selective reduction of the IUGR fetus was proposed as the last resort prior to pregnancy termination. This decision was supported by literature findings suggesting the strategy of placental separation and placental blood flow cessation towards reducing the release of placental antiangiogenic factors that are responsible for the pathogenesis of preeclampsia [36]. Following a thorough informed consultation, patient acknowledged the risks of the procedure-related complications, including amniotic leakage, vaginal bleeding, co-twin death and abortion [37,38]. The embryo with IUGR (Embryo 2) was reduced on the 19th week of gestation and the procedure was successfully performed, without any complications. Selective reduction was performed by a senior consultant with specialty in fetal medicine. The procedure was performed under aseptic conditions with continuous ultrasound guidance. Local anesthetic, 10 mL of 1% lignocaine, was implemented prior to inserting a 15 cm 20 G needle (Cook Ob/Gyn, Spencer, IN, USA) into the fetus’ left ventricle. Following aspiration of 1 mL of fetal blood to confirm correct placement of the needle, strong potassium chloride (15%, 20 mM/10 mL; Phoenix Pharma Ltd., Gloucester, UK) was injected [39].

In the following days, the patient’s clinical condition improved rapidly and impressively. The headaches and nausea were successfully subdued and the patient’s blood pressure was adjusted (mean BP 130/80 mmHg). The thorough follow-up included close monitoring entailing blood pressure evaluations 4 times on a daily basis, full blood count, assessment of liver function and renal function performed 3 times on a weekly basis, along with fetal heart auscultation assessed on a daily basis until delivery. This investigation, indicated that all biochemical and clinical assessment were within the normal range of values, with the exception of detection of high levels of total proteins in the patient’s 24-h urine samples (mean value 1.5 g/day). However, following literature screening it was suggested that the persistent detection of high levels of total proteins in 24-h urine samples in preeclampsia-even following childbirth-may be detected in 30% of the cases and is considered to be associated with preeclamptic lesions correlated with kidney tissue [40]. The improved clinical condition along with the regulation of blood pressure within the normal ranges-not increasing over the 135/85 mmHg mark-were considered significant findings. The intervention of employing selective reduction proved to be a decision enabling progression of this pregnancy.

On the 30th week of gestation and due to vaginal bleeding, an emergency cesarean section was performed. A 1400 g healthy female infant was successfully delivered and following a 24-day stay in Neonatal Intensive Care Unit, the baby was discharged with a recorded weight of 2980 g. A stage one retinopathy of prematurity was the only pathological sign detected. Following delivery, the patient was not subjected to any medical intervention, while her blood pressure levels were at 115/80 mmHg. Mild leukocyturia was the sole pathological finding in her follow-up examination.

Hospital Ethics Board approved the study in accordance to the Helsinki declaration (132/8/6/2020). The patient received extensive consultation during all stages of the decision-making process and provided oral, as well as, written informed consents with regard to all medical interventions performed. Moreover, the patient provided oral and written informed consent for their data to be employed for research purposes, authorizing the authors to collect all information related to this case report for further analysis. In addition, the patient, acknowledging the scientific merit of the case’s publication- following consultation-provided oral and written informed consent and approved the publication of this case.

## 3. Discussion

The definition of preeclampsia has been thoroughly described and established in literature. In clinical practice, the onset of preeclampsia is considered an extremely critical condition hindering maternal health and jeopardizing pregnancy outcome. Certain international guidelines on the recommended therapeutic approach have been proposed assisting clinicians towards efficiently managing such cases [41,42,43]. On the contrary, early onset preeclampsia- when diagnosed prior to the 20th week mark, recruits empirical management on behalf of the clinicians who are left challenged to efficiently address this. This handful of published studies reporting on these cases indicate the literature gap that reflects the lack of knowledge regarding management, while they highlight employment of empirically based methods, stressing the need for further data [20,21,22,23].

A comprehensive screening of the literature revealed that only six published cases have been so far report on the extremely rare phenomenon of early onset preeclampsia prior to the 20th week of gestation (Table 1). It has been voiced that the development of early onset preeclampsia prior to the 20th week of gestation is usually associated with the occurrence of antiphospholipid syndrome or with partial molar pregnancy with triploidy [16,17,18,19]. The cases where early onset preeclampsia prior to the 20th week mark is diagnosed in the absence of the aforementioned disorders are extremely rare in literature, with only six cases published hitherto [14,20,21,22,23,24]. Data provided from these case reports indicate that early onset preeclampsia prior to the 20th week mark is correlated with the typical risk factors of preeclampsia including advanced maternal age (AMA), nulliparity, multiple pregnancies, chronic hypertension, chronic kidney disease, family history of preeclampsia, previous pregnancies complicated with preeclampsia or other pregnancy hypertensive disorder and underlying pathologies namely thrombotic vascular diseases. Interestingly, in five out of the six case reports renal biopsies were performed indicating an underlying renal dysfunction as in all of them glomerular capillary endotheliosis was observed. In all cases a significant improvement with regard to hypertension and proteinuria was observed following medical termination of the pregnancy.

In the present case report, the authors describe a rare case of preeclampsia diagnosed prior to the 20th week of gestation, in a dichorionic, diamniotic twin pregnancy, that resulted following a double embryo transfer employing donor embryos in the blastocyst stage. Pharmaceutical administration of appropriate medication for treating preeclampsia was opted for, albeit symptoms persisted. Concurrently identifying selective IUGR during the 18th week of gestation added another level of complexity to the management. Despite the specific recommendation for terminating the pregnancy the patient denied the suggested course of treatment namely termination of pregnancy. Instead she expressed her desire to exhaust all possibilities towards ensuring survival of at least one of the two embryos. Selective embryo reduction was performed on the IUGR fetus, resulting to an impressive improvement of the clinical symptoms related to preeclampsia. The subsequent improved clinical status of the patient allowed for the progression of the pregnancy-albeit under strict monitoring-for an impressive further 11 weeks until a preterm delivery was performed on the 30th week due to vaginal bleeding. Following emergency caesarian section, a healthy, appropriate for gestational age (AGA) newborn female infant was delivered. Both placentas were extracted during delivery process. The placenta of the IUGR fetus was totally disaggregated and due to the low quality of the sample no analysis could be performed. With regard to the placenta of the female newborn, no signs of partial mole were observed. The immunologic analysis as well as the genetic tests performed revealed no pathological findings. At this point it should be mentioned that in this case report no renal biopsy was performed. Considering that in the great majority of the similar cases presented in literature, an underlying renal pathology consistent with preeclampsia was observed, the absence of renal biopsy in this case report constitutes a major limitation of this study.

Regarding the management employed in the present case report, and despite the lack of robust data indicating appropriate management of early onset preeclampsia prior to the 20th week mark, the clinical management and approach the authors relied on described in this case report was strictly based on the guidelines set by NICE in 2019 regarding hypertension in pregnancy. Initially, a pharmaceutical regulation of hypertension was attempted by administering an appropriate scheme, while monitoring its efficacy in ameliorating patient’s symptoms. According to the NICE guidelines in 2019, labetalol constitutes the gold standard choice amongst antihypertensive drug for treating pregnant women with preeclampsia. Nifedipine has been proposed as an alternative option if administration of labetalol is not allowed [44]. Additionally to the NICE guidelines, further clinical data indicate that methyldopa may also be considered if labetalol and nifedipine are not considered suitable for prescription. According to patient’s medical history reporting a previous event of allergic asthma in her early adolescence, the option of labatelol was rejected.

In addition, during the publication of the NICE Guidelines in June of 2019, certain nifedipine trademarks were contraindicated in pregnancy by manufacturers, based on the summary of their product’s characteristics. Therefore, the coordinating physician and the clinic opted for administrating methyldopa as the optimal pharmaceutical line of treatment. Nonetheless, considering the mild antihypertensive effect of methyldopa, along with its delayed onset of action, additional actions for regulating patient’s blood pressure levels were required [45].With regard to selective embryo reduction on the IUGR fetus, our team herein formed the hypothesis that since preeclampsia manifests because of abnormal placentation, and IUGR similarly may result due to placental insufficiency, then removing the causative factor from this equation -being the IUGR embryo’s placenta-could present as the solution to treating this preeclamptic pregnancy. Published data indicates that selective IUGR affects 11–25% of twin pregnancies and placenta insufficiency of the less developed embryo plays a crucial role in the pathogenesis of the phenomenon of the twins’ selective IUGR [46,47,48]. Our patient presented with simultaneous IUGR and early onset preeclampsia diagnosis. In light of abnormal placentation presenting with a causative relationship to early onset preeclampsia, perhaps the very fact of the diagnosis of IUGR may serve as the catch-22 phenomenon in this case report.

A comprehensive literature review revealed that only six cases have been hitherto reported on selective embryo reduction for managing twin pregnancies complicated with preeclampsia (Table 2). Hence data on selective twin reduction as a therapeutic strategy for preeclampsia are limited. Audibert et al. reported the first case of treating preeclampsia following selective termination of twins. Nonetheless, this study reported on early onset preeclampsia diagnosed at a later gestational stage than the one described herein. Particularly, selective termination of twins was performed during the 32th week of gestation, which enabled the patient to resume with an uncomplicated pregnancy and deliver on the 38th week [36]. Weg et al., Yu et al. and Heyborne et al. reported one, one and three cases respectively, where successful management for preeclampsia and its symptoms was achieved leading to a subsequent live birth following selective reduction [37,38,49]. As anticipated, cases present with differences regarding maternal age, type of twin pregnancy, gestational age at preeclampsia onset, time for allowing gestational progress from selective embryo reduction to delivery, and gestational age at delivery representing areas to be studied. Hence, additionally to data being restricted to begin with, what is more no further extraction of conclusions are allowed, in light of these discrepancies. What is of value though is that all cases shared common ground as following the selective reduction of the IUGR affected fetus, all clinical manifestations subsided while the patient’s blood pressure was regulated.

A thorough literature search reveals a potential mechanism through which selective twins’ reduction reverses preeclamptic manifestations. It has been hypothesized that cessation of placental blood flow and subsequently, placental separation, reduces the release of placental antiangiogenic factors that sustain the deteriorated placental environment. Based on published data, preeclampsia is considered to be a placental disorder that is accompanied by increased expression of placental antiangiogenic factors, including soluble FMS-like tyrosine kinase-1 (sFlt-1) and soluble endoglin (sENG) [50]. As indicated, women with twin pregnancies have sFlt-1 levels that are twice the value of those corresponding to singleton pregnancies due to the increased placental mass. Both sFlt-1 [51] and sENG are highly expressed in the placentas of IUGR and IUGR-discordant twins [48,52]. Moreover, robust data indicate that sFlt-1 as well as placental growth factor (PlGF) could act as potential biomarkers for diagnosing and monitoring preeclampsia [53,54]. At this point it should be highlighted that data with regard to sFlt-1 and PlGF are not provided in the present study. This is due to the fact that hitherto the examination for these biomarkers is not financially covered by the public health sector being the Greek National Health System and can only be performed privately. The last constitutes a second major limitation of this study.

There is a plethora of data which describe the possible pathogenetic mechanisms behind hypertensive disorders of pregnancy (HDP) in singleton gestations. Summarizing the plausible sequence of events, it is demonstrated that the primary abnormal trophoblastic formation differentiates the anatomy of the functional utero-placental arteries which then leads to abnormal placental perfusion [55,56]. This phenomenon is described by the modern science world as maternal vascular malperfusion (MVM) of the placental bed and includes abnormal spiral artery remodeling along with altered flow dynamics in the intervillous space [57,58]. The injury process results in reduced placental oxygenation and ischemic changes in the placenta which alters the placental production of the pro-angiogenic PIGF-1 and simultaneously triggers the secretion of anti-angiogenic factors such as Sflt1 [59]. The rigorous investigation of the pathogenesis of hypertensive disorders has led to the development of diagnostic biochemical tools in order to make it possible to detect any early manifestation of HDP.

Although these disorders are well-investigated in singleton pregnancies, there is no adequate data to support that twin pregnancies share the same pathogenetic mechanism. On the contrary, the dominant hypothesis for twin pregnancies is that the excessive trophoblastic tissue is the starting point in the placental biochemistry alteration and includes the production of impaired PIGF-1 with increased Sflt1 release [51]. Furthermore, recent data indicate that the correlation between MVM and HDP is less in dichorionic twin pregnancies in comparison to singleton gestations and, undoubtfully, this is a field which requires future research in order to clarify the possible pathogenetic mechanism [60]. Moreover, published data suggest that there is significant difference with regard to placenta formation and placenta genetic constitution in dichorionic twin pregnancies leading to an increased risk of preeclampsia especially when selective IUGR is diagnosed [61,62,63]. However, due to the fact that the placenta of the reduced IUGR fetus was completely disaggregated, no information could be provided from this study. This contribution does not aspire to serve as robust evidence towards linking a dysfunctional IUGR placenta to preeclampsia, as no such conclusions can be safely sourced from observations relying on a case report. Future studies providing robust data are needed in order to elucidate the mechanisms entailed in the possible therapeutic efficiency that selective IUGR embryo reduction could exert in managing cases of preeclampsia.

Employing a tool that will enable early detection of placental disorders such as IUGR has been suggested in literature [64]. Genetic, environmental and epigenetic factors are all equally considered as potential etiopathological mechanisms involved in the onset of placental disfunctions [65]. Focusing on the aspect of epigenetics, RNA transcriptional regulation through miRNAs constitutes an epigenetic mechanism activated by several factors participating by altering placental vascular development. These factors include maternal parameters, namely intrauterine nutrient availability and age, as well as environmental factors namely drug administration and infectious agents [64]. The novel promising tool of miRNAs is introduced to distinguish not only between physiological and pathological events that may occur during pregnancy, but also between various pathologies such as preeclampsia, IUGR, and gestational diabetes all sharing the common finding of placental dysfunction [64,65].

Despite the recommendation for termination of pregnancy as the established method to effectively manage a preeclamptic pregnancy and ascertain mother’s health and safety, the physician, weighting in the strong desire of the mother to maintain the pregnancy opted to perform a selective reduction on the grounds of IUGR. Selective reduction management in this case report represented the effort to balance the desired clinical outcome-being regulation of the patient’s blood pressure to 135/85 mmHg, along with mitigating the preeclamptic complications while enabling progress of the gestation for one fetus abiding by the patient’s desire. Fetal blood circulation inside the placenta is ceased instantly following selective termination [37,66]. However, the placental tissue remains [67] and this may serve as an explanation to the phenomenon of the reduction failing to entirely relieve the patient of placental factors associated with the pathophysiology of preeclampsia [66].

## 4. Conclusions

It is noteworthy that the reported case presents an extremely rare clinical phenomenon of early onset preeclampsia prior to the 20th week of gestation, coupled by another adverse clinical condition of selective IUGR resulting to heightening the severity of the prognosis. The combination of these conditions adds to the rarity of the present case report highlighting the gap in existing published data, and indicating the critical necessity to report on how this case was managed, rendering this study timely and essential. The underlined significance herein is that the justified selective fetal reduction for the fetus with IUGR granted an unexpected and valuable extension of the pregnancy’s development for an additional 11 weeks. It is this very fact that rendered the pregnancy viable and allowed for an otherwise uncomplicated gestation leading to live birth of a healthy infant. The authors acknowledge that case reports lack the robustness required to enable establishment of a claimed optimal approach on the matter, and therefore refrain from concluding on bold statements. Nonetheless, this study uniquely presents an interesting and successful management, while addressing a complex case of early onset preeclampsia diagnosed prior to the 20th week mark, entailed in a twin pregnancy with a fetus diagnosed as IUGR. This is one of a few rare cases in literature reporting on a live birth following diagnosis of early onset preeclampsia prior to 20 weeks of gestation. Due to the lack of consensus and recommended therapeutic protocols, sharing available data that stem from such rare cases with the scientific community is important. An efficient and safe strategy treatment outside the scope of empirical approaches remains the Holy Grail for pregnant women diagnosed with early onset preeclampsia prior to the 20th week mark.

## Figures and Tables

**Table 1:** Literature synthesis of case reports referring to early onset preeclampsia prior to the 20th week of gestation without antiphospholipid syndrome or without partial molar pregnancy with triploidy.

Case	Tanaka et al. [14]	Schena et al. [24]	Maya [23]	Stillman et al. [22]	Imasawa et al. [21]	Hazra et al. [20]
Patient Age (years)	30	44	17	30	35	41
Gravidity (number)	NP	7	0	1	0	NP
Parity (number)	0	2	0	0	0	3
Risk Factor for PE	Nulliparity	AMA, familiar hypertension history, chronic hypertension, previous PE	Nulliparity	Nulliparity, Twin pregnancy	Nulliparity, Twin pregnancy	Previous PE
Gestational age at PE onset (week)	18	16	19	15	15	18
Renal Biopsy	Consistent with PE	Consistent with PE	Consistent with PE	Consistent with PE	Consistent with PE	Not performed
PE Management	Methyldopa and steroids	Labetalol, nifedipine, methyldopa, low-molecular-weight heparin, aspirin, medical termination of pregnancy	Methyldopa, medical termination of pregnancy, hydralazine and magnesium sulfate, nifedipine	Labetalol, nifedipine, Aldomet, medical termination of pregnancy	Methyldopa, nifedipine, heparin, medical termination of pregnancy	Methyldopa, heparin, medical termination of pregnancy, labetol
Maternal outcome	BP normal few weeks following delivery, urinary protein levels normal 6 month postpartum	Normal BP and protein/creatinine ratio 26 weeks postpartum	BP improved	Normal renal function few weeks following pregnancy termination	BP normalized and proteinuria decreased three weeks following pregnancy termination	Normal BP with labetol, normal urine analysis
Perinatal outcome	In utero death, SGA	IUGR, medical termination at 22 week of gestation	Nonviable fetus with no gross congenital abnormalities	Cytogenetic analysis revealed normal fetuses	Female fetuses without gross congenital abnormalities	In utero death, no gross congenital abnormalities

PE: Preeclampsia; NP: Not provided; BP: Blood pressure; SGA: Small for gestational age; AMA: Advanced Maternal Age; IUGR: Intrauterine Growth Restriction.

**Table 2:** Literature synthesis of case reports reported on selective embryo reduction for managing twin pregnancies complicated with preeclampsia.

Case	Weg et al. [49]	Yu et al. [38]	Heyborne et al. [37]	Heyborne et al. [37]	Heyborne et al. [37]	Audibert et al. [36]
Patient Age (years)	NP	NP	46	37	45	32
Parity (number)	NP	NP	0	0	1	0
Type of twin pregnancy	Dichorionic triamniotic	NP	Dichorionic	Trichorionic triplet	Dichorionic	Dichorionic diamniotic
Gestational age at PE onset (week)	16	NP	26	24	16	28
Gestational age at SER (week)	16 (reduction of monocorionic twin)	27	26 (reduction of IUGR twin fetus)	24 (reduction of IUGR twin fetus)	16	32 (reduction of IUGR twin fetus)
Time of delivery (week)	32	29	At term	34	At term	38
Maternal outcome	No symptoms of preeclampsia following SER	No symptoms of preeclampsia following SER	No symptoms of preeclampsia following SER	No symptoms of preeclampsia following SER and labetalol administration	No symptoms of preeclampsia following SER	No symptoms of preeclampsia following SER
Perinatal outcome	Live Birth	Live Birth (pre-term)	Live Birth	Live Birth (pre-term)	Live Birth	Live Birth

PE: Preeclampsia; NP: Not provided; SER: Selective Embryo Reduction; IUGR: Intrauterine Growth Restriction.
